# Integrating SpyCatcher/SpyTag covalent fusion technology into phage display workflows for rapid antibody discovery

**DOI:** 10.1038/s41598-019-49233-7

**Published:** 2019-09-06

**Authors:** Julie K. Fierle, Johan Abram-Saliba, Matteo Brioschi, Mariastella deTiani, George Coukos, Steven M. Dunn

**Affiliations:** 1 Department of Oncology, Ludwig Institute for Cancer Research Lausanne, University of Lausanne, Lausanne, Switzerland; 2 Department of Oncology, Ludwig Institute for Cancer Research Lausanne, Lausanne University Hospital and University of Lausanne, Lausanne, Switzerland

**Keywords:** Antibody fragment therapy, Proteins

## Abstract

An early bottleneck in the rapid isolation of new antibody fragment binders using *in vitro* library approaches is the inertia encountered in acquiring and preparing soluble antigen fragments. In this report, we describe a simple, yet powerful strategy that exploits the properties of the SpyCatcher/SpyTag (SpyC/SpyT) covalent interaction to improve substantially the speed and efficiency in obtaining functional antibody clones of interest. We demonstrate that SpyC has broad utility as a protein-fusion tag partner in a eukaryotic expression/secretion context, retaining its functionality and permitting the direct, selective capture and immobilization of soluble antigen fusions using solid phase media coated with a synthetic modified SpyT peptide reagent. In addition, we show that the expressed SpyC-antigen format is highly compatible with downstream antibody phage display selection and screening procedures, requiring minimal post-expression handling with no sample modifications. To illustrate the potential of the approach, we have isolated several fully human germline scFvs that selectively recognize therapeutically relevant native cell surface tumor antigens in various *in vitro* cell-based assay contexts.

## Introduction

Over the past 20 years display technologies have become established as robust and powerful approaches for the *in vitro* isolation of protein and nucleic acid-based ligands with utility for both clinical and non-clinical applications^1–3^. However, despite considerable advances in library construction and binder enrichment strategies, certain aspects of display-based discovery technologies remain inefficient and potentially rate limiting. Among these is the need to generate recombinant, functionally immobilized antigens or antigen-fragments to sustain the needs of multiple *in vitro* selection and screening activities. Usually, antigen material is provided chromatographically purified, often with oligo-histidine or IgG Fc affinity tags present. In this format, it can be passively adsorbed directly to plastic well or tube surfaces, or biotinylated to permit essentially stable binding to streptavidin-coated matrices. The latter allows for significantly greater flexibility with regard to selection procedures and for this reason is generally favoured.

Biotinylation of antigens can be carried out using either chemical or enzyme-catalyzed approaches^4,5^, however these procedures often require antigen-specific optimization and, together with the requirement for purified protein, represent a significant inertia for rapid and multiple-throughput library selection projects. Approaches to circumvent this bottleneck have sought to exploit the native prokaryotic biotinylation apparatus, such that fusion of antigens to the biotin carboxyl carrier protein (BCCP) or to minimal biotin acceptor peptides (BAPs) have become established approaches for the *in vivo* production of naturally biotinylated recombinant molecules in *E. coli*^6,7^. Eukaryotic cells co-transfected with both BAP-tagged antigens and bacterial biotin ligase (BirA), and supplied with exogenous biotin have likewise been shown to efficiently produce biotin-BAP-antigen fusions in both the cytosolic and secreted compartments^8–11^.

Despite its apparent attraction for display technology workflows, *in vivo* biotinylation is still subject to certain process drawbacks. Key among these are variable and inconsistent levels of biotinylation, inefficient biotinylation of proteins secreted to the media from bacterial hosts, and the hydrophobic character of biotin which could, in principle, lead to a reduction in the solubility of certain proteins and promote undesirable aggregation.

Alternative possibilities for direct antigen production and capture are provided by the classical protein fusion tags such as glutathione *S*-transferase^12^, carbohydrate polymer-binding proteins^13–15^, and the Fc fragment of IgG^16,17^. However, although matrix-based purification (including bead capture) using these tag systems is well established, they each suffer from shortcomings and potential incompatibilities with library display and selection workflows. Common reported issues relate to the size of the tag itself (26 kDa for GST; 40 kDa for maltose binding protein, MBP; 50 kDa for dimerizing Fc) and to the aggregation of fusion proteins due to oxidation-induced oligomerization (GST)^18^. Similarly, certain engineered enzyme fusion tags, despite their potential for direct stable covalent capture via catalytic cycle trapping, are typically large (eg. 34 kDa for Halotag7) which may explain why they have not been widely adopted for display technologies^19–21^.

Decisions regarding the production of complex protein antigens can heavily influence the outcome of *in vitro* antibody and antibody-fragment discovery projects. Whereas reagent or ‘tool’ binders intended for use in denatured protein assays (eg. immunoblots) can often be successfully isolated by using arrays of synthetic peptides, such molecules frequently fail to recognize native antigens on cells that generally present folded spatial or discontinuous surface epitopes rather than linear peptides. Reported strategies that seek to produce antigens in a high-throughput generic manner often fail to replicate adequately the structural characteristics of many native antigens, often due to the absence of eukaryotic post-translational and/or redox modifications important for conformational folding and/or function^19^. In this regard, we opted to investigate the recently reported SpyCatcher/SpyTag (SpyC/SpyT) ligand pair, which, in common with Halotag, results in an irreversible covalent bond between the interacting partners^20,21^. Unlike Halotag however, SpyC/SpyT is a ‘split’ protein system which, following association, is stabilized via the formation of an unusual side-chain amide bond. The reaction is reported as being rapid and insensitive to media, buffer composition, detergents, pH, temperature, and oxidation status, and with no requirement for metal ion addition^21^. Additionally, the relatively small size of the SpyC domain (~12.5 kDa) could be considered advantageous for phage panning with a reduced likelihood of propagating tag-directed ‘background’ binders.

Here we report the utility of SpyC/SpyT as a convenient and robust capture tool for the acceleration of antibody fragment discovery by phage display.

## Results

### Preliminary evaluation of *E. coli* for the production and secretion of mammalian extracellular protein domains fused to Spycatcher

**Figure 1:**
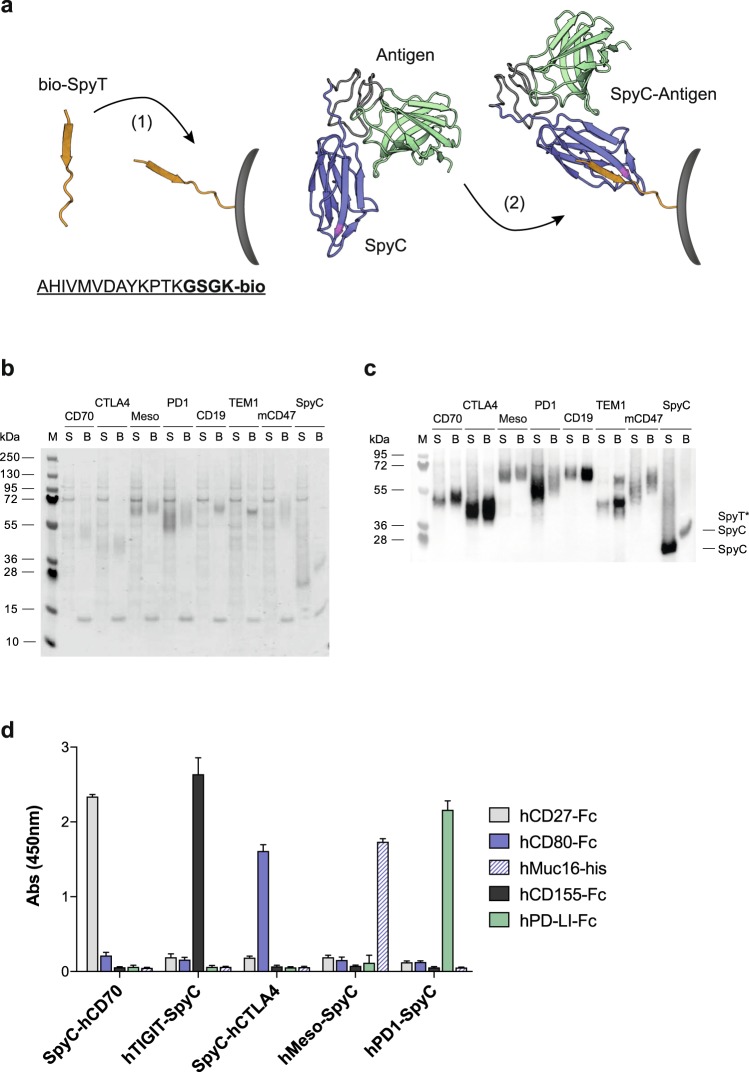
Production and functional evaluation of mammalian extracellular antigen-SpyCatcher fusions. (**a**) Schematic representation of the direct capture and immobilization (dCI) of SpyC-antigens via surface-bound bSpyT. (**b**,**c**) Mammalian cell expression and direct capture of representative SpyC-antigen fusions (see Table 1) on bSpyT-loaded magnetic beads. (**b**) Coomassie-stained SDS-PAGE (reducing). The insert-less pSTEVe49 vector cassette expresses the free HA-tagged SpyC (*far right*). (**c**) Anti-HA tag ECL Western staining of recombinant SpyC-antigens expressed in HEK culture supernatants (‘S’), and following selective covalent enrichment on SpyT beads (‘B’). Covalent immobilization to SpyT on the beads results in a shift to a higher MW corresponding to the mass of the peptide adduct. Samples were run on separate gels and interspersed with irrelevant samples, but were processed in parallel. Full-length gel and blot images can be found in Supplementary Fig. S2. (**c**) ELISA illustrating functional recognition of selected immobilized dCI SpyC-antigens by cognate recombinant ligands.

We initially sought to confirm that the CnaB2-derived SpyC domain could function as an effective C-terminal fusion partner under typical prokaryotic expression conditions and, importantly, that the desired fusions could be secreted to the media at levels sufficient to allow subsequent solid-phase capture and enrichment. To avoid potential steric interference between the upstream protein of interest and the SpyC tag, we chose to include the majority of the parental CnaB2 N-terminal amino acid sequence, and incorporated tandem epitope tags as an additional spacer moiety (pSTEVe16, Supplementary Fig. S1). For solid-phase immobilization, we synthesized a biotinylated minimal SpyT (bSpyT) peptide with an additional tetra-peptide ‘linker’ extension and a C-terminal lysine-biotin to allow a pre-coating of streptavidin-coated beads or plate wells (Fig. 1a).

As experimental test ‘antigens’ we first selected extracellular domain components (ECDs) of the human CD3 complex assembled in a single chain format, and a series of corresponding anti-CD3 scFv antibody fragments. CD3-SpyC fusions, expressed and secreted by *E. coli* TG1 cells, could be captured specifically on bSpyT-loaded streptavidin coated beads and plate wells (Supplementary Fig. S1). Importantly, soluble CD3εδ and CD3εγ SpyC-fusions were bound by UCHT1, an anti-CD3 mAb known to interact with a non-conserved epitope found only in CD3 heterodimers and not in misfolded or monomeric CD3ε (Supplementary Fig. S1)^22,23^. Similarly, the anti-CD3 scFv-SpyC fusions, expressed and captured on a bSpyT-coated surface, were shown to retain their expected T cell activating functionality (Supplementary Fig. S1d).

Collectively, these observations suggested that the direct capture and immobilization (dCI) of SpyC-protein fusions from clarified bacterial media is efficient and that fused antigens can retain their functional structure.

### Pipeline production of SpyC-antigens for antibody phage display using a small-scale mammalian expression system

Following our validation studies in *E. coli*, we next constructed vector cassettes (pSTEVe20/38/49; Supplementary Fig. S1) appropriate for use in a mammalian transient expression system based on the HEK293-6E host cell line. We selected a diverse test panel of mammalian membrane protein ECD fragments representing established and prospective therapeutic target antigens for solid and haematological malignancies, and differing in size, charge and structural complexity (Table 1). Antigens were fused N- or C-terminally to SpyC and targeted to the secretory pathway by appending to a common synthetic signal peptide. From SDS-PAGE and Western blotting of supernatants from 2 ml cultures we were able to show that 30 of the 32 antigen fragments (93%) could be expressed as fusions and secreted, and that of these, 26 (81%) could be directly captured from media supernatants on bSpyT-loaded magnetic beads for subsequent phage display experiments (Table 1, Fig. 1b,c,d and Supplementary Fig. S2). Encouragingly, a sub-panel of dCI SpyC-fused antigens were shown to retain specific recognition for their well-described physiologically relevant ligands (Fig. 1d), confirming that SpyC-fusions, expressed and secreted from mammalian host cell lines, can retain native structural characteristics.

**Table 1:** Antigen ECD fragments evaluated as SpyC fusions in the current study, and selected physical characteristics.

Antigen ECD	Key Architecture	PTM	MW/pI	Vector	Expression	Capture
*SpyC*	β; FBR; IgS	—	12.5/4.6	e49	✓	✓
CD3εδ	β; IgC	2 N-gly (δ)	21.5/4.6	e20	✓	✓
CD3γε	β; IgC	2 N-gly (γ)	22.7/5.0	e20	✓	✓
EpCAM	α/β; Thy1	3 N-gly	27.3/5.6	e38	✓	✗
				e49	✓	✓
TEM8	α/β; VWFA	3 N-gly	33.1/5.4	e38	✓	✗
TEM8 (*nSP*/cΔ)	α/β; VWFA	2 N-gly	23.3/5.3	e38	✓	✓
TEM1 (cΔ)	α/β; CTL; Sus; EGFLc	~4 O-gly	37.6/4.6	e20	✓	✓
TEM1 (cΔ)*	α/β; CTL; Sus; EGFLc	~9 O-gly	49.4/4.6	e20	✓	✓
TEM1 (nΔ)	Mucin-like domain	~15 O-gly	14.0/7.2	e49	✓	✓
CEACAM1	β; IgV, IgC	~20 N-gly	31.8/4.7	e38	✓	✓
CEACAM5 (CEA)	β; IgV, IgC	~28 N-gly	32.3/5.6	e38	✓	✓
CEACAM6	β; IgV, IgC	~12 N-gly	31.4/5.1	e38	✓	✓
CD70	β/coil^‡^; TNF-L	2 N-gly	17.4/8.9	e38	✓ (weak)	✓ (weak)
				e49	✓	✓
Mesothelin	α; ATR	3 N-gly	34.3/5.0	e38	✓	✓
ROR1	β/coil^‡^; IgC; Krn; Frz	4 N-gly	42.5/5.4	e38	✓	✓
ROR2	β/coil^‡^; IgC, Krn, Frz	3 N-gly	41.5/6.2	e38	✗	✗
TNFR2	β/coil; tnfr-cr	4 O-,2 N-gly	25.3/7.0	e38	✓	✓
CD19	β/coil; IgC	5 N-gly	29.8/7.2	e38	✗	✗
				e49	✓	✓
BCMA	β/turn; tnfr-cr		5.9/7.6	e38	✓	✓
CTLA4	β; IgV	2 N-gly	13.5/4.6	e38	✓	✓
				e49	✓	✓
PD1	β; IgV	4 N-gly	16.3/8.8	e38	✓	✓
Tigit	β; IgV	2 N-gly	13.2/4.8	e38	✓	✓
TIM3	β; IgV	1 O-,1 N-gly	20.2/6.6	e38	✓	✓
TIM1	β; IgV; mucin stalk	x O-,4 N-gly	29.1/7.0	e38	✓ (*Het*)	✓ (*Het*)
				e49	✓ (*Het*)	✓ (*Het*)
HER2-CTF611	N/A (peptide)	1 N-gly	4.7/4.4	e38	✓	✓
PSMA	α/β; PA; pM28	10 N-gly	79.5/6.4	e49	✗	✗
SIRPα	β; IgV		12.9/8.0	e38	✓	✓
SIRPα*	β; IgV	2 N-gly	12.9/9.0	e38	✓	✓
CD47	β; IgV	5 N-gly	13.5/5.4	e20	✗	✗
				e38	✓	*NT*
CD47*	β; IgV	6 N-gly	13.7/5.1	e20	✓	✓
FRα			25.1/8.3	e38	✓ (weak)	✗
LAG-3	β; IgV, IgC		46.1/9.5	e38	✓ (weak)	✗
EGFRvIII	β; RLD; FL; GFR4	8 N-gly	38.3/6.5	e38	✓	✓

Unless otherwise indicated, the recombinant ECDs are human and terminate proximal to a predicted TM domain/GPI anchor. Indicated *potential* post-translational modifications are derived from curated UniprotKB features and predictive O-glycosylation tools (NetOGlyc 4.0 Server)^43^. Fusions were assessed for both expression and bead-capture by SDS-PAGE and anti-HA tag ECL Western blotting. Domain folds and motifs: FBR, fibronectin binding repeat; IgS, Ig-superfamily; thy1, thyroglobulin-like type 1; IgV, Ig-like V-type; IgC, Ig-like C2-type; VWFA, Von Willebrand factor type A; CTL, C-type lectin-like; Sus, Sushi; EGFLc, EGF-like calcium binding; ATR, ARM-type repeats; Krn, kringle; Frz, Frizzled; TNF-L, Tumor Necrosis Factor-like; tnfr-cr, TNFR cysteine rich repeats; PA, protease associated; pM28, peptidase M28; RLD, Receptor L-domain; FL, furin-like cysteine-rich; GFR4, growth factor receptor 4 domain. *Murine sequence; ^‡^prediction by PSIPRED v3.3; *nSP*, native signal peptide; cΔ, ECD truncation with C-terminal (membrane proximal) deletion; nΔ, ECD truncation with N-terminal (membrane distal) deletion; *Het*, expression and/or capture product appears very heterogeneous by SDS-PAGE; *NT*, not tested.

### dCI SpyC-fusion antigens represent suitable targets for scFv antibody discovery by phage display

**Figure 2:**
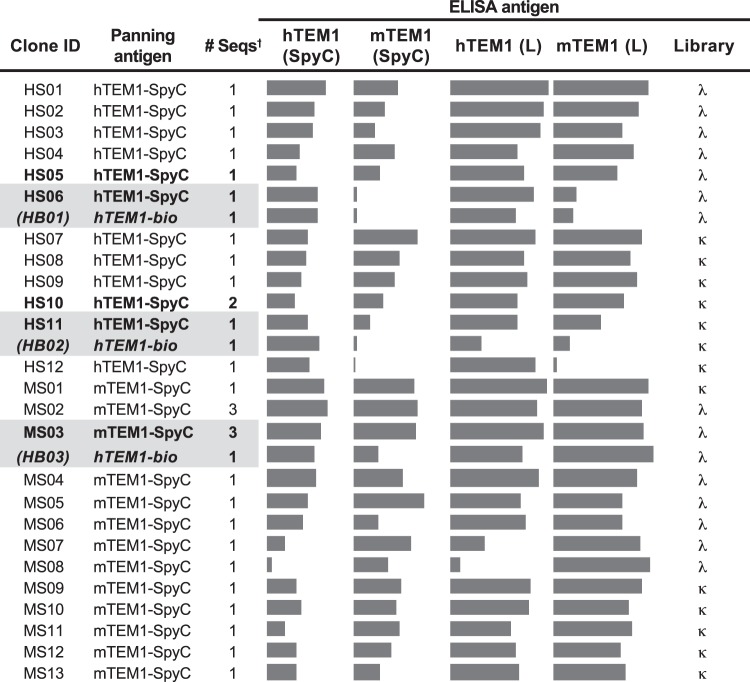
Phage display versus TEM1 antigen format. Raw ELISA profiling data for 30 randomly selected scFv hit clones isolated from the dCI h/mTEM1-SpyC antigen panning experiments. Bar length reflects the relative ELISA signal strengths for human and murine TEM1-SpyC and recombinant, CHO-cell derived and purified TEM1 full-length ECD antigens (L). ELISAs were conducted separately and independently for the TEM1-SpyC and TEM1 (L) antigens. Identical clone sequences obtained from 21 randomly picked hTEM1-bio R2 ELISA hits are shaded. SpyC antigens were captured via dCI on prepared streptavidin-bSpyT plate well surfaces whereas purified recombinant TEM1 (L) was immobilized on plate wells by passive adsorption.

We next sought to evaluate how dCI SpyC-fusion antigens performed in scFv phage display experiments alongside a classically purified antigen. We therefore expressed an ECD fragment of human tumor endothelial marker 1 (TEM1, CD248, Endosialin; N-terminal mature 353 residues) in HEK cells and purified the protein by Ni-NTA chromatography. The protein was subsequently biotinylated enzymatically, quality-controlled by band-shift gel assay, and dialyzed into PBS prior to its immobilization onto streptavidin magnetic beads. In parallel, we produced SpyC fusions in HEK cell culture media for dCI using truncated h/mTEM1 ECDs (N-terminal mature 353 and 460 residues respectively). Using large, fully human and essentially germline naïve scFv libraries (CHV101_DMκ and CHV101_DMλ), we applied two rounds of phage display selection against the three bead-immobilized antigens. An examination of selection metrics (Table 2) suggested strong enrichment for binders at round 2 using both antigen formats, resulting in significant ELISA-positive hit frequencies for both lambda and kappa libraries. Random sequencing of only modest numbers of re-arrayed hits (30 for the combined h/mTEM1-SpyC panel and 41 for the hTEM1-bio) revealed considerable clonal diversity within the h/mTEM1-SpyC panel with ~83% of clones being represented by a unique sequence. This compares favourably with the number of ‘uniques’ identified from the hTEM1-bio sequencing (~90%, data not shown). Interestingly, three of these clone sequences (Fig. 2) were also found to occur in the hTEM1-bio panel, suggesting that the two antigen formats present shared or equivalent epitopes. Further, all hit clones emerging from the dCI TEM1-SpyC outputs also recognized the corresponding full length ECDs of human and mouse TEM1 (668 and 680 mature ECD residues respectively) that were produced in a rodent cell line (CHO) and purified via Ni-NTA chromatography. Importantly, the relative binding activities of these clones towards the human and murine TEM1-SpyC antigens appeared to be broadly conserved for the corresponding classically produced antigens (Fig. 2). Taken together, the data demonstrates that an arbitrary selection of diverse unique hit clones isolated by panning against SpyC-formatted TEM1 antigens can recognise TEM1 produced and purified using traditional procedures, implying that a substantial level of structural ‘equivalence’ is preserved between the SpyC-fusion and traditional antigen formats. Importantly, very few enriched clones were found to recognize the SpyC domain (<5% of screened clones isolated against h/mTEM1) indicating that, despite being of bacterial origin, the small SpyC domain does not appear to be excessively immuno-dominant when using naïve, native content antibody libraries. Supplementary Table 1 summarizes the frequencies of clones binding isolated SpyC in screened outputs from several SpyC-fusion phage panning experiments conducted without active SpyC deselection (ie. subtraction).

**Table 2:** R1 and R2 input and output panning metrics illustrating respective library enrichments and primary ELISA hit-rates for both TEM1-bio and TEM1-SpyC antigens.

Library		hTEM1-bio		hTEM1-SpyC		mTEM1-SpyC	
		λ	κ	λ	κ	λ	κ
R1	Input (phage; CFU)	7.4 × 10^12^	5.3 × 10^12^	7.4 × 10^12^	5.3 × 10^12^	7.4 × 10^12^	5.3 × 10^12^
	Output (CFU)	1.4 × 10^6^	1.1 × 10^6^	5.6 × 10^5^	6.3 × 10^5^	4.4 × 10^5^	3.2 × 10^5^
R2	Input (phage; CFU)	1.6 × 10^11^	1.2 × 10^11^	1.2 × 10^11^	1.4 × 10^11^	2.4 × 10^11^	1.9 × 10^11^
	Output (CFU)	2.3 × 10^9^	1.7 × 10^9^	3.0 × 10^9^	2.6 × 10^9^	5.3 × 10^9^	4.1 × 10^9^
	Enrichment Factor*	**7.6 × 10** ^**4**^	**6.8 × 10** ^**4**^	**3.3 × 10** ^**5**^	**1.6 × 10** ^**5**^	**3.7 × 10** ^**5**^	**3.6 × 10** ^**5**^
R2	ELISA hit rate (%)^‡^	43	25	56	42	68	44

CFU, colony-forming units; *(R2 Output/Input)/(R1 output/input), ^‡^signal threshold >5x background.

In addition to traditional plate-based ELISA screening, we also employed crude SpyC-antigen supernatants in bead-based multiplexed assays with the aim of acquiring higher information content for selection outputs. Fluorescently barcoded streptavidin bead multiplexes, pre-loaded with four to six distinct dCI SpyC antigens were used to screen phage display output clone supernatants for specific and/or cross-reactive antigen binding. By incorporating the dCI methodology into a ‘no wash’ direct read 384-well protocol on an iQue Screener Plus instrument (IntelliCyte), we were able to greatly accelerate primary output specificity screening (Supplementary Fig. S3). Furthermore, we were able to use this approach to successfully identify improved affinity matured clone sequences enriched against the same cognate dCI SpyC-antigen in a high-stringency phage display selection experiment (Supplementary Fig. S3).

### Secreted SpyC-antigen fusions support the isolation of scFvs that recognize native endogenous cell epitopes and have downstream functional utility

**Figure 3:**
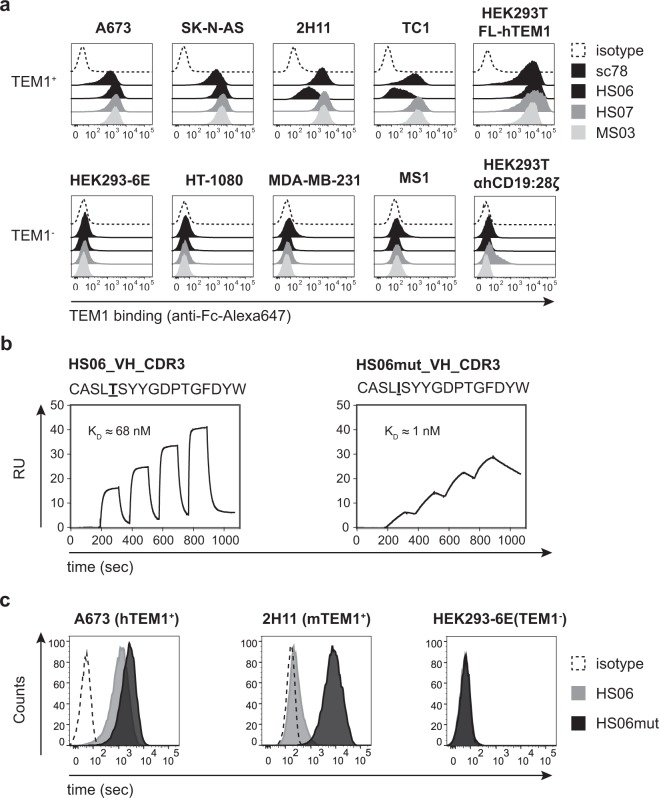
Characterization of anti-TEM1 clones isolated by dCI SpyC-antigen selection. (**a**) Binding of reformatted and purified scFv-Fc (hIgG1) clones (2 μg/ml) to human and murine cell lines. A673, human Ewing’s sarcoma; SK-N-AS, human neuroblastoma; 2H11, murine tumor vascular endothelium; TC1, murine lung adenocarcinoma; HEK293-6E, human embryonic kidney; HT-1080, human fibrosarcoma; MDA-MB-231, human mammary carcinoma; MS1, murine pancreatic islet endothelium. Additionally, binding of anti-TEM1 scFv-Fc clones to HEK293T cells transiently transfected with native FL-hTEM1 and an irrelevant surface protein (αhCD19:28ζ CAR) control is also shown. (**b**) SPR monovalent affinity determination of parental clone HS06 (*left*) and an affinity-matured variant, HS06mut (*right*). hTEM1-SpyC has been used as the immobilized ligand and monovalent HS06/HS06mut monovalent BiTE as the soluble analyte, with concentration ranges of 100, 50, 25, 12.5 and 0 nM for HS06, and 5, 2.5, 1.25, 0.625 and 0 nM for HS06mut. (**c**) Binding of HS06mut variant scFv-Fc (0.1 μg/ml) to endogenous human (A673) and murine (2H11) TEM1^+^ cell lines.

To investigate whether scFvs isolated against dCI SpyC-fusions could recognize endogenous cognate antigens on appropriate target cells, we reformatted three representative anti-TEM1 clones as scFv-Fc (IgG1) fusions alongside a previously described anti-TEM1 antibody, sc78^24^. All of the clones expressed well in our transient HEK293-6E system (100-200 mg/l) and, following purification by Protein A affinity chromatography, all antibodies were seen to stain HEK293T cells transfected with full-length hTEM1, as well as several cell lines previously established as positive for endogenous TEM1, but not lines negative for TEM1 (Fig. 3a). One clone, HS06, showed clear preference for recognition of the human A673 and SK-N-AS TEM1^+^ lines with significantly reduced binding to the 2H11 and TC1 TEM1^+^ murine lines. During downstream characterization of HS06, we were also able to confirm that SpyC fusions have convenient and practical utility for affinity determination using surface plasmon resonance (SPR). The simple addition of free bSpyT peptide to crude HEK expression supernatant containing secreted hTEM1-SpyC, followed by rapid buffer exchange via spin column, allowed the efficient capture of the resultant covalent complex on a streptavidin-coupled Biacore chip. Rapid single-cycle kinetic binding data obtained for purified monovalent H06 scFv yielded a modest double-digit nanomolar affinity (Fig. 3b), characteristic of many antibody clones isolated from natural germline repertoires that have not undergone somatic hyper-mutation and clonal expansion. Encouragingly, reference KD data obtained using a commonly employed anti-Fc capture step to immobilize HS06 scFv-Fc as the ‘ligand’, a full-length (FL) recombinant hTEM1 ECD ‘analyte’, and multi-cycle kinetics, were shown to be comparable (Supplementary Fig. S4). To explore whether primary scFv hits arising from SpyC-fusion panning were suitable for subsequent entry into classical lead optimization experiments, we mutated the HS06 scFv by error-prone PCR to generate a library of ~10^9^ variants and panned this library sequentially against both immobilized mTEM1-bio and hTEM1-bio in order to select for increased-affinity variants with h/mTEM1 cross-reactivity. A strongly enriching clone (HS06mut) containing a single VH CDR3 amino acid substitution was recovered and its affinity determined using both hTEM1-SpyC and purified full-length hTEM1 ECD. Remarkably, this single point mutation was shown not only to confer a ~70 to 80-fold improvement in raw KD towards human TEM1 in both formats (Fig. 3b and Supplementary Fig. S4), but also to possess appreciable cross-reactivity to mTEM1 (Fig. 3c). Taken together, our data validates TEM1-SpyC as a flexible reagent for the isolation and early characterization of functional anti-TEM1 molecules using highly streamlined dCI methodology.

We subsequently sought to demonstrate that SpyC-fusion antigens would be suitable for more challenging discovery projects; for example, the isolation of scFvs intended to neutralize specific receptor-ligand interactions. To investigate this, we chose the murine signal regulatory protein alpha (SIRPα):CD47 interaction as a test case. After confirming the correct folding and efficient capture of the mCD47 ECD SpyC-fusion, we panned the libraries against bead-captured mCD47-SpyC and then screened the resultant binder clones by dCI competition ELISA to identify scFvs that specifically blocked the interaction between dCI mCD47-SpyC and an Fc-fused version of the CD47 ligand, SIRPα. Encouragingly, scFv clones with neutralizing activity could be readily isolated from the CHV101_DM libraries and, following reformatting to murine IgG2a, a representative candidate (MS115) was further characterized alongside two neutralizing rodent hybridoma mAbs^25^. Clone MS115 was shown to compete effectively with the binding of SIRPα for CD47-SpyC and to retain its neutralization activity using a His-tagged and purified mCD47 antigen sourced from a commercial vendor (Supplementary Fig. S5). Critically, MS115 recognized endogenous mCD47 present on a murine tumor cell line but did not stain the corresponding knock-out (Supplementary Fig. S5). Hence, dCI SpyC-antigen selection and screening is compatible with the rapid isolation of target-selective neutralizing antibodies.

### dCI SpyC-antigens are suitable targets for the selection of functional scFv warheads for immunotherapy applications

**Figure 4:**
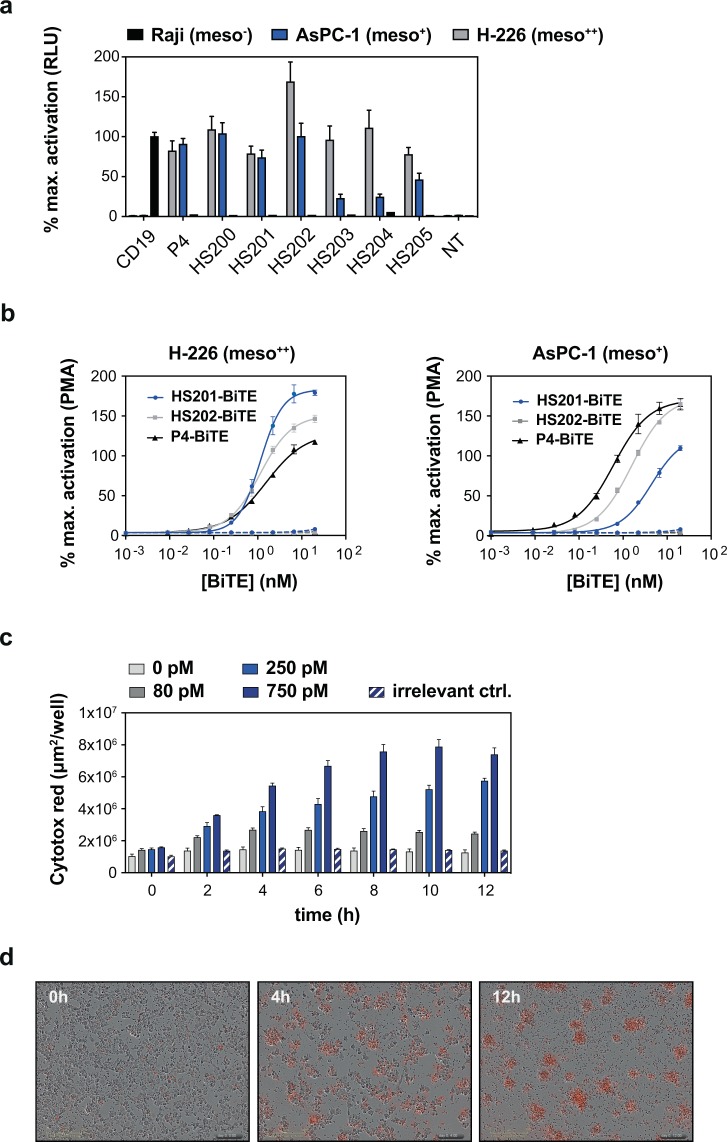
Functional T cell bridging and target cell engagement by anti-mesothelin scFvs isolated from dCI selection experiments. (**a**) Induction of NFAT-driven expression of luciferase in Jurkat reporter cells transduced with anti-mesothelin CARs employing different scFv clones. Luciferase activity was measured after 24 h of co-culture with mesothelin expressing target cells (H-226, AsPC-1) or meso-negative Raji B cells. CD19 and P4 CARs are included as positive controls for CD19 and mesothelin respectively. NT, non-transformed control. (**b**) Induction of NFAT-driven expression of luciferase in Jurkat reporter cells in the presence of H-226 (*left*), and AsPC-1 (*right*) target cells and soluble T cell engagers. Dashed lines represent baseline stimulation in the presence of engager molecule and HEK293-6E mesothelin-negative cells. (**c**) Mesothelin specific T cell engager constructed from clone HS201 redirects primary human CD8^+^ T cells to kill H-226 tumor target cells. Image-based acquisition of Cytotox Red fluorescence reports the extent and real-time kinetics of killing. Baseline killing was determined against irrelevant meso-negative A673 cells using 750 pM engager. (**d**) Time-dependent killing of H-226 cells in the presence of 2 nM HS201 engager by primary human T cells (Pan-T purified; 14 days post isolation). Dead cell clusters emit red fluorescence.

Aside from classical antibody targeting applications, scFv ‘warheads’ have also demonstrated considerable utility in recruiting and re-directing the exquisitely potent killing functions of cytotoxic T cells towards tumor-associated cell-surface targets. The two principle therapeutic paradigms, namely chimeric antigen receptors (CARs) and soluble bispecific ‘engagers’, both facilitate T cell-mediated killing by ‘bridging’ target and T effector cells followed by the transmission of activation signals from intracellular CD3 and co-receptor signalling components. We therefore sought to evaluate the utility of the dCI SpyC-antigen format for the rapid discovery of functional CAR scFv ‘warhead’ panels. To this end, we chose the mature ECD of human mesothelin as a relevant immunotherapeutic target^26,27^. Following panning of the CHV101_DM libraries against dCI mesothelin-SpyC, and the subsequent identification of binder clones in both dCI mesothelin-SpyC primary ELISA and secondary flow cytometry assays, six scFvs of interest were reformatted into a generic 2nd generation CAR comprising a CD28 spacer, transmembrane (TM) domain and cytosolic domain fused to CD3ζ immunoreceptor tyrosine-based activation motif (ITAM) signalling elements. The resultant CAR panel was transfected into an engineered Jurkat reporter cell line to assess CAR-mediated NFAT activation in response to target cell lines known to express endogenous cell surface mesothelin. All six clones were shown to trigger a strong induction of CD3 ITAM-mediated NFAT signalling in the presence of mesothelin-expressing H-226 tumor cells (Fig. 4a). The magnitude of these signals compared favourably with those observed for a control CAR armed with the clinically validated murine anti-CD19 FMC63 scFv in the presence of CD19-expressing Raji cells, and with a published anti-mesothelin human scFv (P4)^28,29^. In the presence of AsPC-1 tumor cells, which are known to express lower levels of surface mesothelin, three of the six clones yielded substantially less NFAT reporter activation, probably reflecting the relatively modest monovalent affinities of these primary non-optimized scFv binders (data not shown).

To explore further the potential of the anti-mesothelin clones originating from the SpyC dCI selections, we constructed soluble T cell engagers from two of the novel scFvs showing promising CAR signalling activity, together with P4. Unlike classical BiTEs which employ two monovalent scFvs linked in tandem^30^, our final molecules were bivalent for the anti-mesothelin warheads and monovalent for a humanized anti-CD3. Similar to the CAR format, the three resulting T cell engagers were shown to trigger potent NFAT-mediated activation of the Jurkat reporter line in a cell target and dose-dependent manner (Fig. 4b). Interestingly, while the HS201 and HS202 clone engagers displayed similar activation kinetics to the P4 benchmark clone against H-226 meso-high cells, clear differences in sensitivity were evident against meso-moderate AsPC-1 cells, with the HS201 engager being the most discriminatory. Extending the study to cell killing confirmed that this experimental T cell engager molecule could efficiently redirect primary human T cells to kill H-226 tumor cells *in vitro* in a time and concentration dependent manner (Fig. 4c,d). Collectively, the results indicate that our dCI procedure using small scale, SpyC-fusion supernatants can be employed successfully for the rapid and cost-effective isolation of functional scFv ‘warheads’ for a range of potential applications, including cancer immunotherapy.

## Discussion

In the current study, we sought to develop a minimal, cost-effective and generic solution to native mammalian cell-surface antigen production that could be easily integrated into a typical scFv antibody phage-display discovery workflow. With this in mind, we decided to evaluate the reportedly robust and irreversibly associating SpyC/SpyT ligand pair in combination with a widely used human cell-line protein production host using modular episomal expression vector tools.

To-date, the SpyC/SpyT interaction has been reported principally in the context of defined molecular assembly and imaging applications^31–35^. Following the elucidation of the original 2-component system, the inventors further engineered the SpyC module to function as an effective uncoupled peptide ligase for head-to-tail oligomerization of proteins of interest tagged with both SpyT and a partner peptide (SpyK) containing the corresponding reactive lysine^36^. However, to the best of our knowledge, the evaluation of SpyC/SpyT as a practical tool for antibody phage display-based molecular discovery applications has so far not been reported.

In this work, we synthesized chemically a SpyT peptide with a biotin handle to allow the controlled pre-coating of immobilized streptavidin matrices with a high molar density of SpyT ligand to favour avid surface capture of SpyC-fusion proteins present in crude media supernatants. Although more cost-effective and efficient than expressing and purifying large stocks of recombinant biotinylated SpyC protein, a principle concern was that the use of the larger bacterial SpyC component for the antigen fusion partner would prove incompatible with efficient expression and/or secretion from cellular hosts. However, we observed that a codon-optimized SpyC sequence preceded by an immunoglobulin signal peptide was exported efficiently by HEK293-6E cells. Our subsequent findings confirm that the SpyC module tolerates fusion to many diverse mammalian cell-surface extracellular fragments/domains, and retains its functional integrity (as shown by efficient and selective capture on SpyT-surfaces) following its secretion by HEK293 cells in either an N- or C-terminal fusion orientation (Fig. 1 and Table 1). By employing an optimized transient transfection protocol and a high-productivity HEK293 cell line, sufficient SpyC-antigen fusion material can be generated from small culture volumes to support multiple parallel and diverse discovery activities comprising several dCI selection and screening rounds.

Interestingly, our broader observations demonstrate that even poorly expressing fusions can yield successful outcomes. For example, the secreted concentration of murine CD47-SpyC was estimated by ECL-Western analysis to be in the region of only 1 μg/ml and below our limit of detection for visible SDS-PAGE dye staining in crude expression media. However, SpyT-bead enrichment from 2 ml of media proved adequate for efficient and robust phage-display clone enrichment, mirroring the selection performance of SpyC-antigens expressing at far higher levels and allowing the isolation of functional clones (Supplementary Fig. S5).

Collectively, our scFv phage display screening data gathered to-date suggests that the bacterial SpyC tag sequence is only modestly immunogenic as a fusion partner using our IgM/IgD-derived libraries – even in the absence of a typical solid-phase deselection step (Supplementary Table 1). Conveniently, SpyC has an additional advantage for display applications in that a single point mutation destroys its capability to form the covalent iso-peptide bond with SpyT^21^. Hence, purified SpyC[mut] can presumably be employed as an effective soluble library subtraction reagent or can be exploited in its fusion form as a soluble competitor during off-rate selections.

In principle, we would anticipate that SpyC/SpyT dCI methodology could be readily integrated into any binder discovery platform that employs a solid-phase capture for enrichment, for example ribosome display, as well as phage display using non-immunoglobulin or synthetic library scaffolds. It could also be adapted for approaches that enrich for binders using flow cytometry-based sorting of particles, for example yeast, mammalian, bacterial and aptamer particle display technologies. For these cases, SpyC-antigen expression supernatants could be directly ‘charged’ with synthetic SpyT conjugates containing either biotin or appropriate bright dye adducts prior to incubation with library particles and sorting. Finally, the direct interfacing of dCI from small-scale cultures with automated magnetic bead handling would support applications requiring high-throughput scaling of selections^37^.

In conclusion, we have extensively tested the practical utility of SpyC/SpyT and shown it to be an attractive and convenient tagging/immobilization tool for *de novo* antibody hit-discovery selection, screening and characterization, circumventing the classical requirement for traditional antigen purification/processing. We have confirmed its general compatibility with i) mammalian soluble expression and secretion, and ii) the direct capture and immobilization of fusion antigens from crude cell media. The provision of covalent anchorage for antigen targets by SpyC/SpyT dCI, such that surface complexes can survive the prolonged incubations and extensive washing steps typically applied during stringent selection protocols, has permitted the successful isolation of several functional antibody candidates recognizing diverse therapeutic targets.

## Materials and Methods

### Prokaryotic expression and secretion of SpyC-fusion proteins

The nucleotide sequence of the CnaB2-derived SpyCatcher (SpyC) domain was synthesized (GeneArt, Thermo Fisher Scientific) with reference to the sequence of Genbank accession JQ478411.1 (amino acids 28–136) and cloned into a pUC119-derived prokaryotic expression vector (pSTEVe16) containing a periplasmic secretion signal. The protein of interest was cloned N-terminal to the SpyC domain, separated by tandem myc/his or HA/his epitope tags. The amino acid sequences of the extracellular region of the human CD3ε, δ and γ chains were obtained from UniProtKB (http://www.uniprot.org/)^38^ and assembled as single-chain heterodimers using a 28 amino acid flexible spacer. The amino acid sequences of anti-CD3 antibody variable domains were extracted from the following published patents: UCHT1 (US8519100), OKT3 (Blinatumomab; WO2005052004), Pasotuxizumab (WO2008119567A2), Solitomab (US8076459B2), Foralumab (US 20060177896A1). A murine CD3-specific scFv was constructed from the sequenced 145-2C11 hamster mAb^39^. Genes were synthesized in scFv format and cloned into pSTEVe16.

*E. coli* TG1 cells (Lucigen, #60502) were electroporated with the relevant plasmids and grown overnight on agar prepared with 2TY medium supplemented with 2% glucose and 100 μg/ml ampicillin (2TYAG). Colonies were inoculated into 96-well plates containing 2TYAG and cultures were grown overnight at 30 °C. For protein expression, 4 μl of overnight culture was used to inoculate 170 μl Terrific Broth (TB) containing 0.1% glucose and 100 μg/ml ampicillin. The cultures were grown in 96-well PP U-form microplates (Greiner, #650201) with shaking at 30 °C for 3–4 h until reaching an OD600 of 0.6. Protein expression was induced by adding 20 μl per well of 1 mM Isopropyl-β-D-thiogalactopyranoside (IPTG; Merck, #420322) to achieve a final concentration of 100 μM. Expression was allowed to continue for 16–18 h at 30 °C with shaking at 750 rpm, 70% humidity before harvesting of media for subsequent analysis.

### Eukaryotic protein expression and purification

Extracellular domain (ECD) fragments of human and mouse membrane antigens were synthesized (GeneArt, Thermo Fisher Scientific) based on sequences and predicted topologies obtained from UniprotKB. Gene fragments were fused N- or C-terminally to vector-encoded SpyC housed in pTT-based mammalian episomal expression vectors^40^. All vectors contained an identical semi-synthetic signal peptide (pSTEVe20/38/49 constructs). Selected scFv candidates were typically either sub-cloned into a pTT-based vector containing a vector-encoded human IgG1 constant region to produce scFv-Fc fusion proteins, reformatted into modular pTT-based IgG heavy and light chain vectors for co-transfection and production of assembled IgGs, or fused to anti-CD3 UCHT1 domains to generate secreted T cell engagers.

Recombinant protein was produced using the mammalian HEK293-6E/pTT transient expression system (National Research Council of Canada; obtained under licence). HEK293-6E cells were grown in Freestyle F17 medium (Thermo Fisher Scientific, #A13835) containing 4 mM GlutaMAX (Life Technologies, #35050061), 0.1% Pluronic® F-68 (Life Technologies, #24040032) and 25 μg/mL G418 (Life Technologies, #10131019) at 37 °C, 5% CO_2_ and 120 rpm. For transfection, the DNA was mixed with FectoPRO (Polyplus, #116-010) transfection reagent in F17 medium without supplements, according to the manufacturer’s instructions. After five days of protein expression, cultures were subjected to low speed centrifugation and the media collected. Samples could be used immediately for direct capture and immobilization (dCI) experiments or snap-frozen and stored at −80 °C until required.

ScFv-Fc fusions and IgG molecules were purified from clarified expression media using a HiTrap™ MabSelect column (GE Healthcare, #11003494), followed by extensive dialysis against phosphate-buffered saline (PBS). His-tagged T cell engagers were purified by IMAC chromatography using a HisTrap™ Excel column (GE Healthcare, #17-3712-05). The peak monomer fractions were pooled and buffer-exchanged into PBS using a Superdex 200 Increase 10/300 GL preparatory grade column (GE Healthcare, #28-9909-44). The 6xhis-tagged variants of human or murine TEM1 ECD containing a C-terminal biotinylation sequence were purified using a HisTrap™ excel column followed by site-directed enzymatic biotinylation using purified BirA^41^. Following confirmation of biotinylation by avidin gel shift assay^42^, the proteins were buffer exchanged into PBS supplemented with 0.1% BSA and stored at −80 °C.

Transient transfection of HEK293T cells with the FL-hTEM1 cDNA ORF (extracted from Genbank RefSeq NM_020404.3) and an irrelevant membrane-localised control ORF (anti-hCD19 2^nd^ generation CAR construct) utilized the pTagGFP2-N CMV promotor vector (Evrogen, #FP192). Briefly, HEK293T cells were detached and plated in 6-well plates at 10^6^ cells/well in a volume of 4 ml. Recombinant plasmid DNA (4 μg) was combined with 400 ml serum-free DMEM and 6 ml Turbofect reagent (Life Technologies, #R0532), and incubated for 20 min at RT before being added dropwise to the plated cells. Transfected cells were maintained at 37 °C, 5% CO_2_ under a humidified atmosphere for 48 h.

### Direct capture and immobilization (dCI) of secreted SpyC-fusion proteins for downstream library selections

For solid-phase immobilization of SpyC-fused antigens, we synthesized a C-terminally biotinylated SpyT peptide (bSpyT) with a 4-amino acid ‘spacer’ extension (>90% purity; Protein and Peptide Chemistry facility, UNIL). Streptavidin-conjugated magnetic beads (80 μl; M-280 Dynabeads; Life Technologies, #11206D) were suspended in 1 ml of PBS containing 1 μM of bSpyT peptide and incubated overnight at 4 °C or 1 h at room temperature (RT) with gentle rotation. After 3 × 1 ml wash steps in PBST (PBS + 0.1% Tween-20) and 1 × 1 ml wash step in PBS, the coated beads could be stored at 4 °C until required or incubated directly with 2 ml of SpyC-fusion expression supernatant for 2 h with gentle rotation at RT. The antigen-coated beads were washed 4x with PBST and 1x in PBS and generally used immediately for dCI evaluation and phage display experiments.

### SDS-PAGE and Western blotting

To elute bound protein, SpyC-antigen coated beads were re-suspended in 1x LDS buffer (NuPAGE; Life Technologies, #NP0007) with 10% reducing agent (NuPAGE; Life Technologies, #NP0009) and boiled at 95 °C for 10 min. The beads were pelleted by centrifugation and the eluted protein fraction was separated on a Novex 4–12% Bis-Tris Gel (Life Technologies, #NP0321) for 38 min at 200 V. Separated protein fractions were visualized by Coomassie Blue staining (InstantBlue; Expedeon, #ISB1L) or transferred to nitrocellulose membranes (iBlot2; Thermo Fisher Scientific, #IB23001). After blocking with 5% milk/PBST, the membranes were incubated with an HRP-conjugated anti-HA tag antibody (Thermo Fisher Scientific, #PA1-29751), diluted 1:1000 in PBS containing 1% BSA. Membranes were washed four times in PBST and HA-tagged protein was detected with an enhanced chemi-luminescent (ECL) HRP substrate (Thermo Fisher Scientific, #34080), followed by image acquisition using a Fusion FX imaging system (Vilber).

### Selection of human germline scFvs by phage display using SpyC-fusion proteins

All phage display selections were carried out using two large, fully human scFv libraries (CHV101_DMκ and CHV101_DMλ; SMD, unpublished) constructed from the peripheral blood of 120 healthy volunteer donors (Service de Transfusion, Epalinges, Switzerland). Briefly, IgD/IgM VH, and Vκ/Vλ domain repertoires were amplified from cDNA prepared from mRNA affinity-purified from enriched CD19^+^ cells, using a newly designed primer panel. Amplicons were pooled, purified and cloned sequentially (Vκ/Vλ followed by VH) into the gIII display cassette of a newly designed phagemid vector (pCHV101; SMD, unpublished). A bacterial library of ~2 × 10^10^ colonies was generated following electroporation into *E. coli* TG1 cells. Phage library rescue was performed according to standard published procedures and single-use phage aliquots were stored in a stabilization buffer at −80 °C.

Streptavidin magnetic beads pre-coated with bSpyT and coated with SpyC-antigens (or coated directly with purified and biotinylated h/mTEM1 ECD) were used as solid-phase targets for phage display selections. The scFv phage libraries were first blocked with PBST containing 2% skimmed milk, 1% BSA for 30 min at RT, and then incubated for 30 min with streptavidin beads coated only with SpyC domain to subtract (‘de-select’) non-specific or SpyC-specific binders. Subsequently, blocked and de-selected phage particles were transferred to tubes containing similarly blocked SpyC-antigen beads, and incubation was continued at RT for 1 h. Non-binding phage were removed by 5 × 1 ml washes with PBST followed by 1 × 1 ml PBS. Typically, the stringency of selection was increased at the second round by transferring the 5 × 1 ml PBST washed beads into a Falcon tube containing 50 ml PBST and allowing lower affinity phage to passively dissociate over 20 min prior to rapid magnetic capture and final washing with 1 ml 1x PBS. Bound phage were eluted from the beads with 200 μl of 20 μg/ml trypsin (Sigma Aldrich, #T1426) in PBS for 30 min at 37 °C (stationary). Eluted phage were allowed to infect minimal medium-grown *E. coli* TG1 cells grown to an OD600 of 0.4–0.5 in 10 ml of 2TY medium supplemented with 2% glucose (2TYG) for 1 h at 37 °C (stationary). The infected cells were collected by centrifugation at 4000 rpm (RT), re-suspended in 3 ml 2TYG, and plated on 2TYAG agar with incubation at 30 °C for 18–20 h. Colonies were scraped from plates and the cells stored frozen in 2TYG containing 15% glycerol pending further rounds of phage rescue and selection. Phage rescue between rounds was performed according to standard protocols using M13KO7 helper phage (Life Technologies, #18311019) added at a MOI of 5:1. Secreted phage were collected and purified by two rounds of PEG/NaCl precipitation according to standard protocols, and stored as frozen, single-use aliquots in a stabilization buffer.

Clones were cultured for primary screening by picking individual colonies into 2TYAG liquid medium, growing until turbid and then inoculating cells into supplemented TB medium for the induction of protein expression as described above.

### Affinity maturation of anti-TEM1 clone candidates

Selected scFv clones were subjected to random mutagenesis across the whole scFv, using error-prone PCR with the Diversify PCR random mutagenesis kit (Takara, #630703). The scFv was amplified in one, two or three subsequent rounds of PCR with 25 cycles in the presence of 640 μM MnSO4 and 40 μM dGTP in order to generate variants with low, intermediate and high mutational load. Mutated scFv library DNA was cloned into pCHV101 and electroporated into *E. coli* TG1 cells to generate libraries of ~10^9^ colonies. Phage particles displaying the mutagenized scFv libraries were rescued and PEG/NaCl-precipitated before being used in high-stringency affinity maturation selections against either purified bio-TEM1 (‘classical’ approach) or TEM1-SpyC (dCI approach). In order to enrich for high-affinity, cross-reactive binders towards both human and murine TEM1 ECD, the first round of selection was performed against bead-immobilized bio-hTEM1 and the second round against bio-mTEM1. Both rounds included competition (‘off-rate selection’) with 200 nM free unlabelled antigen and an extended high-volume washing step of 50 ml PBST (30 min for R1 and overnight for R2). For the dCI approach, employed using the SpyC-hTEM1(Δn) fusion target, R1 beads were loaded with HEK expression supernatant diluted 1:10 in PBS with stringent washing following phage binding (6 × 1 ml PBST, 2 × 50 ml PBST (20 min), 1 × 1 ml PBS). For R2, the expression supernatant was diluted further (1:50) and the stringency increased with an additional 2 × 50 ml PBST (20 min) washes. For all experiments, random colonies were picked from the R2 selection output and sequenced to assess clone integrity and diversity prior to the initiation of screening.

### Enzyme-linked immunosorbent assay (ELISA)

For screening of scFv clones (and variant molecules) by dCI SpyC-antigen ELISA, wash steps were performed using 300 μl PBST dispensed from a BioTek 405 automatic plate washer. Nunc Maxisorp 96-well plates (Thermo Fisher Scientific, #442404) were coated with 100 μl of 10 μg/ml Neutravidin (Life Technologies, #31000) in PBS over-night at 4 °C, washed 3x with PBST and incubated with 100 μl of 1 μM bSpyT peptide in PBS for 1 h at RT with gentle agitation. After blocking in 5% skimmed milk/PBST for 1 h, wells were washed 3x with PBST and 100 μl SpyC-antigen expression supernatants (typically diluted 1:10 in PBST + 1% BSA for mammalian expression, or blocking buffer for bacterial expression) were added to allow covalent capture by the bound bSpyT. Incubation was at RT for 1.5 h. Wells were washed 4x with PBST and 100 μl blocked scFv culture supernatants added. Wells were washed 4x with PBST and binders were detected using a primary recombinant anti-myc tag antibody (derived from parental mAb clone 9E10, in-house) and a horseradish peroxidase (HRP) conjugated goat anti-mouse IgG antibody (Sigma Aldrich, #A9917). The colorimetric read-out was developed with TMB substrate reagent (Biolegend, #34029) and stabilized with 2N sulfuric acid. Absorbance was measured at 450 nm and 620 nm on a BioTek Synergy plate reader. ELISAs were performed in parallel against both cognate antigen-SpyC and non-fused SpyC in order to eliminate hits to the latter.

CD3-SpyC single-chain heterodimers were detected using 1 μg/ml of a murine anti-hCD3 antibody (clone UCHT1, Thermo Fisher Scientific, #MA1-80044) followed by a HRP-conjugated anti-HA tag antibody (Thermo Fisher Scientific, #PA1-29751; diluted 1:5000), or with UCHT1-scFv displaying phage particles revealed using an M13 antibody HRP conjugate (Sigma Aldrich, cat. 27-9421-01). For evaluation of cognate ligand binding to captured SpyC-antigens, purified bivalent hFc(IgG1)-fusions (hCD27, hCD80, hCD155, hPD-L1) or his-tagged monomer (hMuc16) were purchased from R&D Systems. Ligands were diluted to 2 μg/ml in PBST + 1% BSA and incubated for 1 h at RT. After washing, bound Fc-fusions and bound Muc16 were detected with goat anti-human IgG Fc polyclonal HRP conjugate (Sino Biologicals, #SSA001; 1:5000 dilution) or anti-His tag HRP conjugate (Genscript, #A00612; 1:1000 dilution) using TMB as substrate.

### Multiplexed bead-based no-wash screening assay using SpyC-fusion proteins

For screening of scFv clones by mix-and-read bead multiplex assay, SpyC-fused antigens in expression supernatants were covalently captured on fluorescently barcoded streptavidin-coated beads (Spherotech yellow beads, #SVFB-2552-6K) using the dCI protocol described above. The antigen-coated bulk bead populations were combined and blocked in assay buffer (PBS/2%BSA/0.1% PBST/2 mM EDTA/0.02% sodium azide) for 30 min on ice. Multiplexed beads (10 μl) were added to wells of a 96-well plate, followed by an equal volume of scFv-containing *E. coli* TG1 expression supernatants diluted 1:5 or 1:10 in assay buffer. After incubating on ice for 30 min, bound scFvs were detected by subsequent addition of 10 μl of an iFluor647-conjugated anti-his tag antibody (GenScript, #A01802-100) at a final dilution of 1:200 in assay buffer. Following a further 20 min incubation on ice, the sample plate was read directly on an Intellicyt iQue TM Screener PLUS instrument (10 s sampling; 1 μl/s). Bead populations displaying different immobilized SpyC-antigens were resolved by their respective barcoding fluorescence intensity (FITC), and scFv binding to each population was assessed by iFluor647-mediated median fluorescence intensity (MFI). Data analysis was performed using Forecyt software (Intellicyt).

### mCD47:mSIRPα competition ELISA screening

96-well ELISA plates were coated as described above with Neutravidin, bSpyT and mCD47-SpyC. Alternatively, plates were coated directly with 0.5 μg/ml commercially available his-tagged mCD47 (Sino Biologicals, #57231-M08H-50). Pre-blocked scFv expression supernatants (100 μl) were added to each well immediately followed by 100 μl of diluted HEK expression supernatant containing recombinant mSIRPα-Fc (hIgG1) fusion produced by transient transfection. Appropriate dilutions of mCD47-SpyC and mSIRPα-Fc that yielded signals within the linear part of the response curve were determined previously by chequerboard ELISA titration. Following incubation for 1 h, the bound mSIRPα-Fc levels were determined by colorimetric detection with goat anti-human IgG Fc polyclonal HRP conjugate (Sino Biologicals, #SSA001; 1:5000 dilution) using TMB as substrate. Neutralizing scFv clones were reformatted as murine IgG2a and purified. Their mCD47:mSIRPα-blocking activity was confirmed in ELISA titration experiments alongside commercially available benchmark rodent mAbs (kindly provided by Irving Weissman lab, Stanford). All competitors were titrated in a 3-fold dilution series starting at 2.5 μg/ml and mixed 1:1 v/v (200 μl final) with in-house produced mSIRPα-Fc HEK supernatant before addition to the plate. After 1 h of incubation, binding of mSIRPα-Fc to plate-bound mCD47 was revealed by goat anti-human IgG HRP-conjugated antibody (Sino Biologicals, #SSA001; 1:5000 dilution) followed by the addition of TMB substrate for colorimetric detection.

### Surface plasmon resonance (SPR)

SPR analysis was performed on a Biacore T200 instrument (GE Healthcare). Experiments involving TEM1-SpyC ligand immobilization used a Series S SA sensor chip (GE Healthcare, #BR-1005-31). Briefly, 1 ml of crude TEM1-SpyC expression media was incubated with 1 μM of bSpyT at RT for 2 h with gentle rotation. The resulting covalent TEM1-SpyC:bSpyT complex was separated from free bSpyT by buffer-exchange into 1x filtered Biacore running buffer (HBS-EP+; 0.01 M HEPES, 0.15 M NaCl, 0.05% Surfactant P20, 3 mM EDTA, pH 7.4; GE Healthcare, #BR-1006-69) using a spin column with a 10 KDa cut-off (Vivaspin 6; GE Healthcare, # 28932296). The biotinylated TEM1-SpyC ligand complex was immobilized on the SA chip at a density of 150 RU. For kinetic analysis, analytes were diluted into running buffer and injections/dissociations carried out at 30 μl/min with data collected in Single Cycle Kinetics mode. For Fc-capture experiments, 10000 RU of AffiniPure Goat Anti-Human IgG (Jackson ImmunoResearch, #109-005-098) were immobilized on a CM5 Series S sensor chip (GE Healthcare, #BR-1005-30) by amine coupling according to the manufacturer’s instructions. Anti-TEM1 scFv-Fc molecules were captured at a target density of 100 RU and analytes were injected and dissociated at 30 μl/min with data acquired in Multiple Cycle Kinetics mode. Surfaces were regenerated between cycles/experiments by injecting 10 mM glycine-HCl, pH 1.5 for 30 s. Corrections for bulk shift and refractive index changes were performed by subtracting the signal of a reference flow cell from the active cell.

### Cell culture

A-673 (ATCC CRL-1598), 2H11 (ATCC CRL-2163), MS1 (ATCC CRL-2279) and HT-1080 (ATCC CCL-121) cells were maintained in Dulbecco’s modified Eagle’s medium (DMEM) supplemented with GlutaMAX and 10% fetal bovine serum (FBS). SK-N-AS (ATCC CRL-2137) and TC1 (ATCC CRL-2934) cells were additionally supplemented with 0.1 mM non-essential amino acids (Gibco, Life Technologies, #11140050). HEK293T (ATCC CRL-11268) cells were cultured in DMEM, 10% FBS and 100 U/ml penicillin/streptomycin (Gibco, Life Technologies, #15140122). MDA-MB-231 (ATCC HTB-26), NCI-H226 (ATCC CRL-5826), AsPC-1 (ATCC CRL-1682), Raji (ATCC CCL-86), A-20 (ATCC TIB-208), A-20_CD47KO (kindly provided by Irving Weissman lab, Stanford) and Jurkat NFAT Lucia cells (Invivogen, #jktl-nfat) cells were cultured in RPMI-1640 Glutamax (Life Technologies, #61870010) containing 10% FBS. A-20 cells were additionally supplemented with 0.05 mM 2-β-mercaptoethanol and Jurkat Lucia cells were maintained under selective pressure using 100 μg/ml zeocin (Invivogen, #ant-zn-1). All cells were maintained at 37 °C, 5% CO_2_ in a humidified incubator and the absence of mycoplasma from all cell lines was confirmed by regular testing (GATC service).

Peripheral blood mono-nucleated cells (PBMCs) were isolated from fresh buffy coats by density centrifugation using Lymphoprep (Axonlab, #1114545). T cells were subsequently extracted by magnetic separation using a human pan-T cell isolation kit (Miltenyi Biotec, #130-096-535) and stimulated with human T cell activator CD3/CD28 beads (Life Technologies, #11161D) and 50 RU IL-2 (Peprotech, #200-02-50UG) for 5 days. After the removal of the beads, primary T cells were further expanded with IL-7 and IL-15 (Miltenyi Biotec, #130-095-367 and #130-095-765) for a further 5–10 days.

### Flow cytometry

Adherent cells were detached using 10 mM EDTA, counted and resuspended in fresh, complete culture medium. All subsequent steps were performed on ice. For each sample, 0.5 × 10^6^ cells were first blocked in FACS buffer (5% FBS in PBS) and then incubated for 1 h with test antibody (typically 1–2 μg/ml) or expression supernatant diluted in FACS buffer. After washing three times with FACS buffer, the following secondary antibodies were added: Alexa Fluor 647 AffiniPure Goat Anti-Human IgG (Jackson Immunoresearch, #109-605-098, 1:200 dilution) or Alexa Fluor 647 anti-mouse IgG (Life Technologies, #A-21236, dilution 1:50). Secondary antibodies were incubated for 30 min and the cells were washed again three times. Immediately before data acquisition, dead cells were stained with 4′,6-Diamidino-2-phenylindole (DAPI, 1:2000 dilution). Data was acquired using an LSR-II flow cytometer equipped with FACSDIVA software (BD Biosciences). Data analysis and plotting were carried out using FlowJo v10 (FlowJo LLC).

### Lentiviral transduction of Jurkat NFAT Lucia cells

Sequences encoding human anti-mesothelin scFvs or an anti-CD19 scFv (FMC63; extracted from Sequence 2, patent US7446179) were linked to a spacer/hinge, transmembrane region and intracellular costimulatory domain derived from the hCD28 costimulatory domain, and to an intracellular hCD3ζ signaling domain. The resulting 2^nd^ generation CAR cassettes were appended to an in-frame monomeric green fluorescent protein ORF (TagGFP2, Evrogen) and cloned into a modified pRRL lentiviral vector (Origin: Didier Trono lab, EPFL). Virus was produced by transient transfection of HEK293T cells using pCMVR8.74 and pMD2.G plasmids for packaging (Origin: Didier Trono lab, EPFL) and Turbofect transfection reagent (Life Technologies, #R0532). Virus-containing supernatant was harvested after 48 h, concentrated by ultracentrifugation and added directly to 5 × 10^5^ Jurkat NFAT Lucia cells. Transduced cells were expanded for 10–14 days before performing functional assays.

### Jurkat NFAT activation reporter cell assays

For CAR and soluble engager assays designed to measure ITAM-mediated ‘Signal 1’ activation responses, 5 × 10^4^ target cells were seeded in 96-well plates and allowed to attach for 20 h. Subsequently, 10^5^ Jurkat NFAT Lucia reporter cells were added to each well. For activation assays using T cell engager molecules, molecules were added in 3-fold serial dilutions, starting from 20 nM. Phorbol myristate acetate (PMA)/ionomycin was included as a positive response control. After 24 h of co-culture, the supernatants were collected and mixed with an equal volume of QUANTI-Luc luciferase substrate (Invivogen, #rep-qlc-1). Luminescence was measured immediately using a BioTec H1MFG Synergy plate reader. For functional analysis of anti-CD3 scFv-SpyC fusions, TG1 *E. coli* expression supernatants were added to Neutravidin/bSpyT-coated 96-well Maxisorp plates and incubated for 2 h at RT to allow covalent capture of fusions. After washing (5x PBST, 2x PBS), Jurkat Lucia reporter cells were added and secreted luciferase determined as above.

### Primary T cell cytotoxicity assay

Specific target cell killing was assessed using real-time kinetic cell imaging (Incucyte system, Essen Bioscience). Target and control cells (1.5 × 10^4^) were seeded in 96-well plates prior to the start of the experiment. When approximately 30% confluency was observed, soluble T cell engager molecules were added as 3-fold serial dilutions, starting from 20 nM. Positive control test wells were treated with 1% Triton X-100. Primary human T cells prepared and expanded as described above were added to the plates (10^5^ per well) to obtain an E:T ratio of approximately 5:1. Cytotox Red reagent (Essen Bioscience, #4632) was added to a final dilution of 1:4000, and resultant cell death was monitored as an increase in fluorescence over time.

## Supplementary information

Supplementary Information

## Data Availability

All data generated or analysed during this study are included in this published article.
