# Supplementary material for: Integrating SpyCatcher/SpyTag covalent fusion technology into phage display workflows for rapid antibody discovery
Source: Sci Rep. 2019 Sep 6;9:12815. doi: 10.1038/s41598-019-49233-7 (PMC6731262; doi:10.1038/s41598-019-49233-7)
Supplement: Supplementary file 1 — Supplementary Information [file 41598_2019_49233_MOESM1_ESM.pdf]

SUPPLEMENTARY INFORMATION

corresponding to the manuscript entitled:

Integrating SpyCatcher/SpyTag covalent fusion technology into phage display workflows for rapid antibody discovery.

AUTHORS

Julie K. Fierle[1], Johan Abram-Saliba[1], Matteo Brioschi[1], Mariastella deTiani[1], George Coukos[1,2] and Steven M. Dunn[1,2]

Author affiliations

[1] Department of Oncology, Ludwig Institute for Cancer Research Lausanne, University of Lausanne, Lausanne, Switzerland.

[2] Department of Oncology, Ludwig Institute for Cancer Research Lausanne, Lausanne University Hospital (CHUV) and University of Lausanne, Lausanne, Switzerland

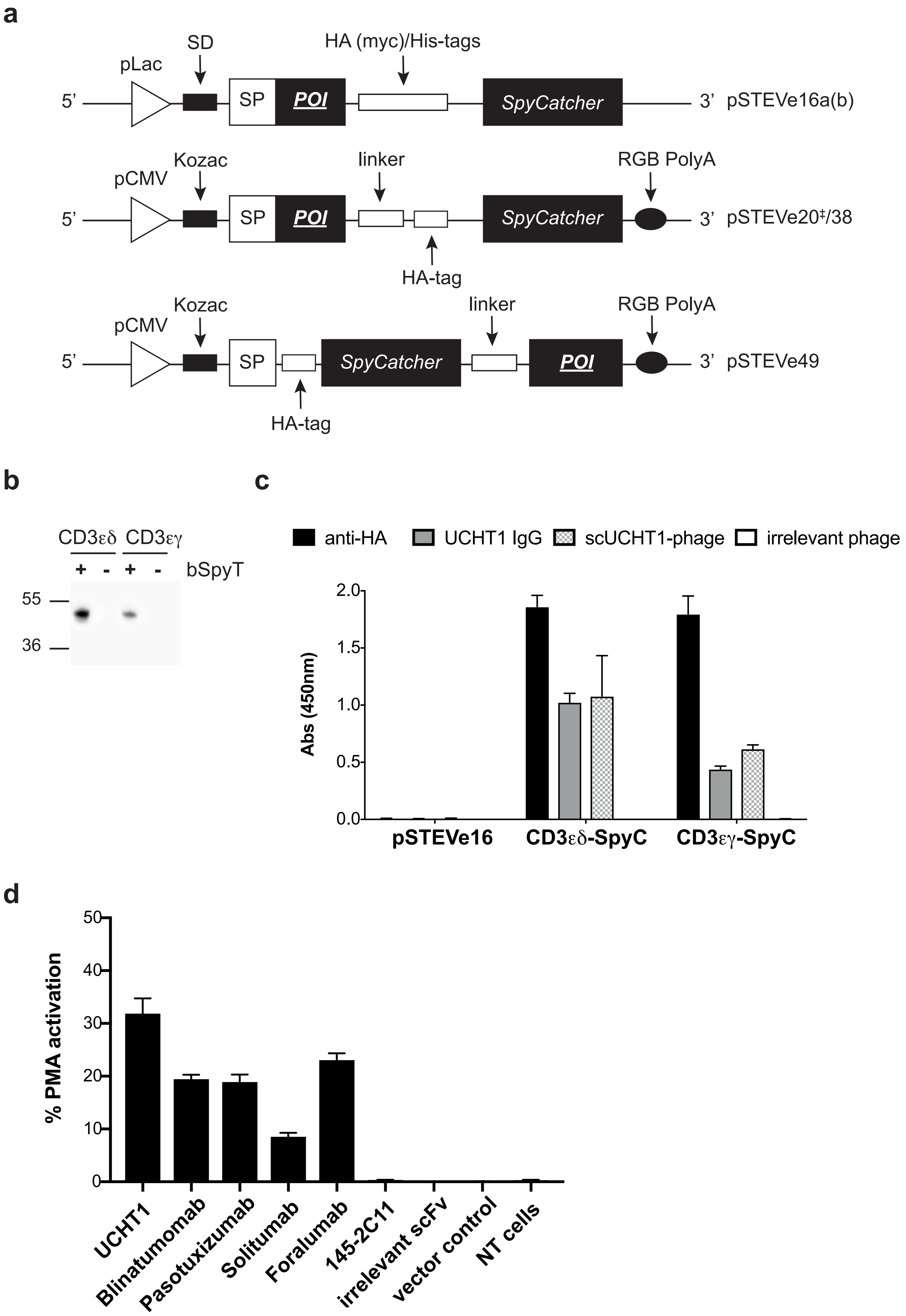
a
SD
HA (myc)/His-tags
pLac
5'
SP
POI
SpyCatcher
3' pSTEVe16a(b)
pCMV
Kozac
linker
RGB PolyA
5'
SP
POI
SpyCatcher
3' pSTEVe20‡/38
HA-tag
pCMV
Kozac
linker
RGB PolyA
5'
SP
SpyCatcher
POI
3' pSTEVe49
HA-tag
b
CD3εδ CD3εγ
+ - + - bSpyT
55
36
c
anti-HA
UCHT1 IgG
scUCHT1-phage
irrelevant phage
Abs (450nm)
2.0
1.5
1.0
0.5
0.0
pSTEVe16
CD3εδ-SpyC
CD3εγ-SpyC
d
% PMA activation
50
40
30
20
10
0
UCHT1
Blinatumomab
Pasotuxizumab
Solitumab
Foralumab
145-2C11
irrelevant scFv
vector control
NT cells

Supplementary Figure S1

Production and functional evaluation of mammalian extracellular antigen-SpyCatcher fusions. (**a**) Prokaryotic and eukaryotic expression vector cassettes used for the production of SpyC-fused proteins. SP, signal peptide; POI, protein of interest, ‡pSTEVe20 is an earlier variant of pSTEVe38 comprising the original non-codon optimized SpyC sequence and a histidine-tag; (**b**) The expression and secretion of single-chain hCD3 heterodimer-SpyC fusions in *E. coli* TG1 using vector pSTEVe16. Bead capture from culture supernatants is dependent on the presence of SpyT on the beads (Western blot detection of intact fusion protein via anti-HA tag ECL). (**c**) Secreted CD3-SpyC heterodimers were directly captured from bacterial media using plate wells pre-coated with Neutravidin and bSpyT, and detected by anti-HA tag and UCHT1 (as both IgG and as a scFv-phage reagent. (**d**) Functionality of a panel of anti-CD3 scFv-SpyC fusions (parental IgG names retained) expressed and secreted from *E. coli*. Fusions captured on Neutravidin/SpyT coated assay plates retain the functional ability to cluster native human cell surface CD3 and stimulate NFAT-driven secretion of luciferase from a Jurkat NFAT reporter cell line. The anti-murine CD3 clone, 145-2C11, does not recognize hCD3 and was included as an additional negative control. NT, non-transformed; PMA, phorbol myristate acetate.

a

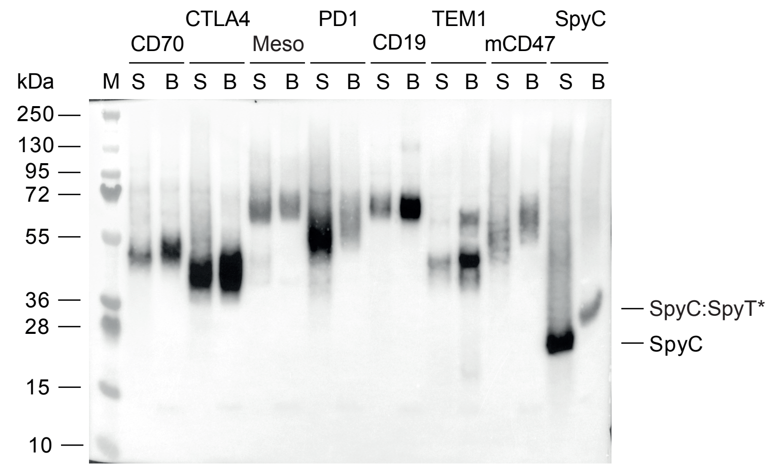

b

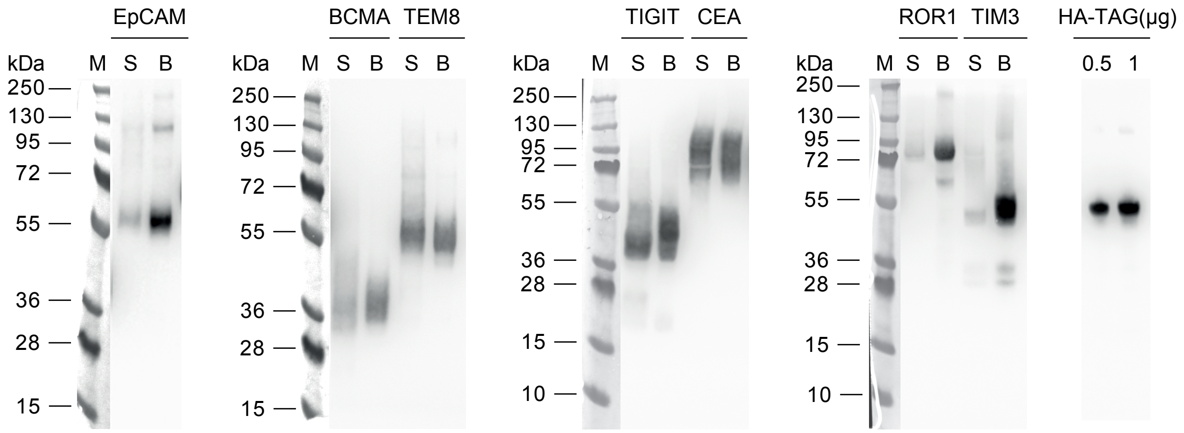

Supplementary Figure S2

Full uncropped image of the Western Blot data shown in Figure 1c and Western Blot analysis of additional SpyC-antigens. (**a**) Full-sized anti-HA tag Western Blot image corresponding to Fig. 1c. Representative SpyC-antigens were expressed in HEK293-6E cells and directly captured from the expression supernatant ('S') onto bSpyT-loaded magnetic beads ('B'). Covalent immobilization to SpyT on the beads results in a shift to a higher MW corresponding to the mass of the peptide adduct. (**b**) Additional SpyC-antigens were captured and analyzed as described above. Samples were run on separate gels in the presence of additional undisclosed samples and controls, but were processed in parallel. Only relevant lanes of full-sized images are shown with their respective size marker.

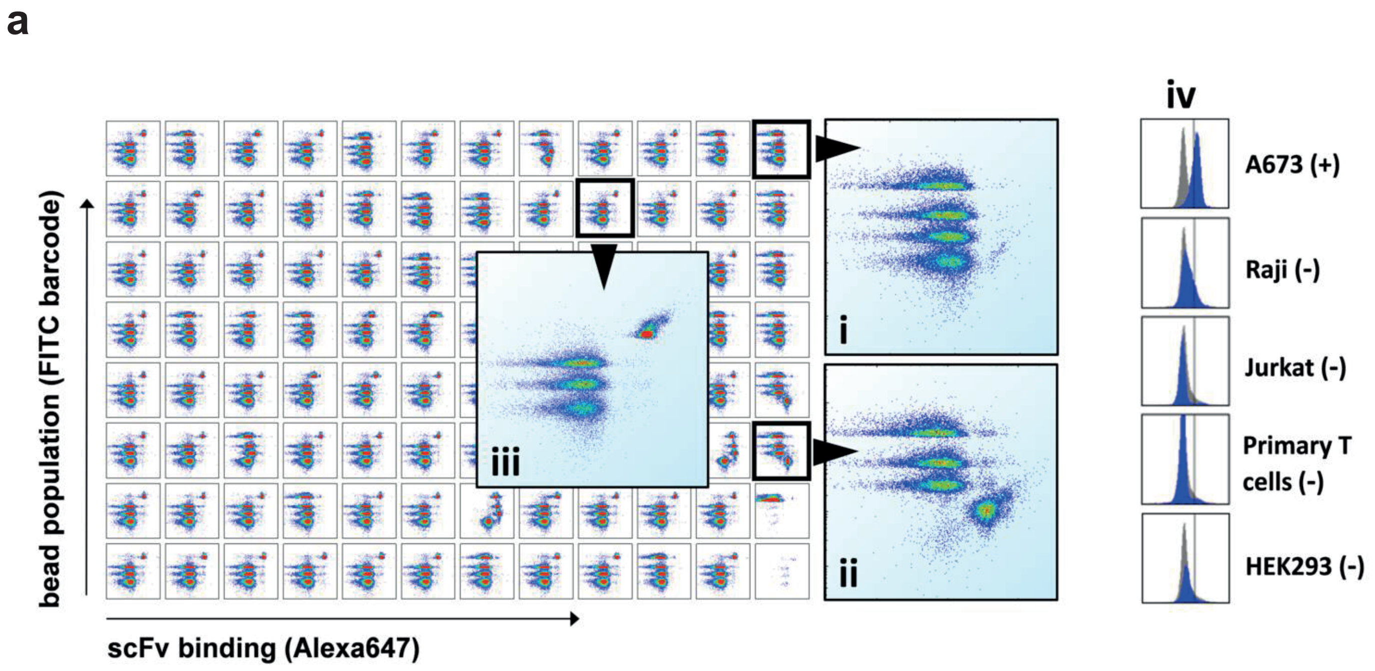

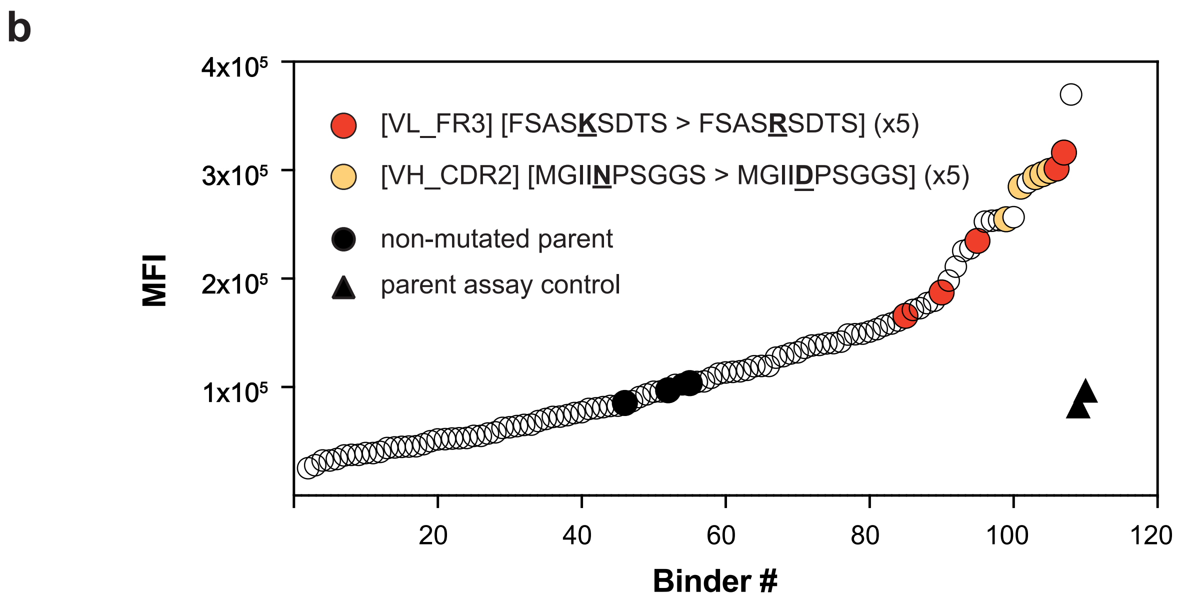

Supplementary Figure S3

Multiplexed, no-wash bead-based primary screening of bacterial scFv clone supernatants from dCI-selected hTEM1(Δn) R2 selection outputs. (**a**) Barcoded streptavidin beads were pre-loaded with bSpyT prior to dCI of the respective antigens from HEK expression supernatants. The resulting 4-plex comprises (from top/brightest): hTEM1(Δn)-SpyC, mTEM1(Δn)-SpyC, SpyC-domain only, hEGFRvIII-SpyC. Expanded panels: (i) irrelevant scFv supernatant (negative control); (ii) scFv specific for EGFRvIII (positive control); (iii) representative screening 'hit' (HS301) selective for hTEM1(Δn); (iv) binding profile of purified HS301 scFv-Fc towards endogenous TEM1^{+} (A673) and TEM1^{-} cell lines (isotype staining control as grey curve). (**b**) Bead-based multiplex MFI distribution plot of binders to SpyC-hTEM1(Δn) coated beads obtained following error-prone mutagenesis of the HS301 parental clone and two stringent dCI selection rounds against SpyC-hTEM1(Δn). Hit threshold was set at 5x background with no binding to mesothelin-SpyC or control SpyC coated beads. Multiple occurrences of enriched point-mutated variants of interest clustering at significantly higher MFI values than the parent are indicated. MFI, median fluorescence intensity.

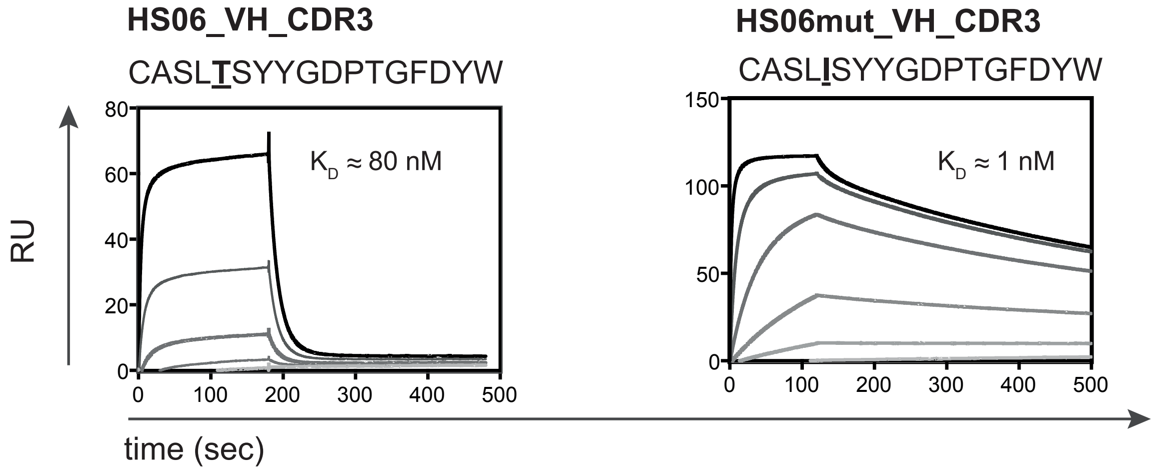

Supplementary Figure S4

SPR monovalent affinity determination of parental clone HS06 (left) and an affinity-matured variant, HS06mut (right), using HS06/HS06mut scFv-Fc as the immobilized ligand. Soluble hTEM1-FL analyte concentration ranges were 100, 50, 25, 12.5 and 0 nM for HS06, and 5, 2.5, 1.25, 0.625 and 0 nM for HS06mut. hTEM1-FL, full-length human TEM1 ECD (NS0 cells; R&D Systems, #7855-CD-050).

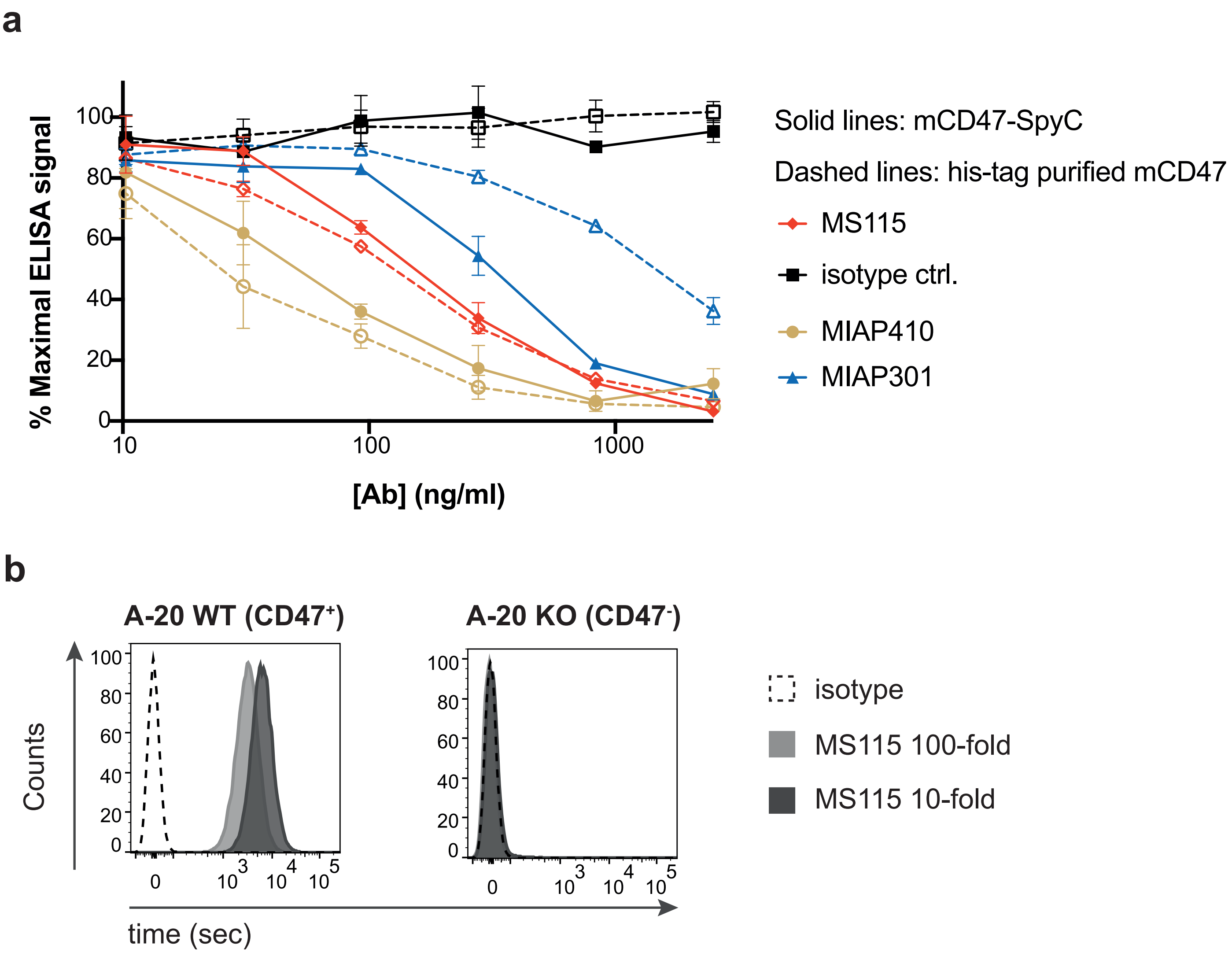

Supplementary Figure S5

Characterization of a mCD47-targeted neutralizing antibody Isolated by SpyC-fusion dCI phage display selection and screening. (**a**) Competitive blocking of the interaction between soluble mSIRPα-Fc and immobilized mCD47. Dashed lines: Commercial his-tag purified mCD47 coated by passive adsorption. Solid lines: mCD47-SpyC captured and immobilized directly from expression media on Neutravidin/bSpyT pre-coated wells. Reference/control antibodies: Black, 9E10 mIgG1 (isotype control); blue, MIAP301 rIgG2a (commercial neutralizing mAb); red, dCI mCD47-SpyC selected clone MS115 mIgG2a; gold, MIAP410 mIgG1 (commercial neutralizing mAb). (**b**) Specific recognition of endogenous mCD47 on murine B cell lymphoma cell line A-20 by clone MS115. Cell binding was assessed by flow cytometry in the scFv-Fc (hIgG1) format at 10- and 100-fold dilution of transient HEK expression supernatant.

Screening Plate	Target Antigen	Library	Round	# Target binders	% Hit Rate‡	# SpyC binders	% SpyC binders*
MP01	**hTEM1-SpyC**	CHV101_DMλ	Rd02	49	55.7	1	4.5
MP02	**hTEM1-SpyC**	CHV101_DMκ	Rd02	37	42.0	1	4.8
MP03	**mTEM1-SpyC**	CHV101_DMλ	Rd02	60	68.2	1	2.3
MP04	**mTEM1-SpyC**	CHV101_DMκ	Rd02	39	44.3	0	0.0
MP07	**hMeso-SpyC**	CHV101_DMλ/κ	Rd02	52	59.1	0	0.0
MP37-40	**hMeso-SpyC**	CHV101_DMκ	Rd02	175	11.9	0	0.0
MP1914	**spyC-hCD19**	CHV101_DMλ/κ	Rd02	23	6.3	4	1.1
MP1915	**spyC-hCD19**	CHV101_DMλ/κ	Rd03	40	10.9	11	3.0
MP4701	**mCD47-spyC**	CHV101_DMλ	Rd02	10	11.9	4	4.8
MP4702	**mCD47-spyC**	CHV101_DMκ	Rd02	1	1.2	1	1.2
MP4703	**mCD47-spyC**	CHV101_DMλ/κ	Rd02	8	9.5	3	3.6

Supplementary Table 1

Primary screening metrics illustrating respective ELISA hit-rates and frequencies of SpyC-reactive clones for selected scFv discovery projects using SpyC-antigens. Hit rates represent the frequency of clones binding to the respective SpyC-antigen in a primary screening. These hits were subsequently re-arrayed and screened for SpyC-reactive clones, the frequency of which is shown. Rd, selection round; ‡ signal threshold > 5x background. * signal threshold > 3x background.
